# Pressure to provide milk among mothers of very low birth weight infants: an explorative study

**DOI:** 10.1186/s12884-024-06315-3

**Published:** 2024-02-13

**Authors:** Isabella Schwab, Till Dresbach, Tim Ohnhäuser, Dirk Horenkamp-Sonntag, Nadine Scholten, Neo-MILK, Andreas Müller, Martin Hellmich, Nicole Ernstmann, Antje Hammer, Friederike Eyssel, Angela Kribs, Juliane Köberlein-Neu, Katharina Lugani, Eva Mildenberger, Jens Ulrich Rüffer, Katja Matthias, Anne Sunder-Plaßmann, Daniel Wiesen, Iris Klein, Melanie Klein, Christoph Rupprecht, Laura Schleich, Olaf Beckmann, Anke Kurz

**Affiliations:** 1 Institute of Medical Sociology, Health Services Research, and Rehabilitation Science, Health Services Research University of Cologne, Faculty of Medicine and University Hospital Cologne, Eupener Straße 129, Cologne, 50933 Germany; 2 Department of Neonatology and Pediatric Intensive Care, Children’s Hospital, University of Bonn, Venusberg-Campus 1, Bonn, 53127 Germany; 3 Techniker Krankenkasse, Healthcare Management, Hamburg, Germany

**Keywords:** Very low birth weight, Maternal stress, Pressure to provide milk, Milk volume, Lactation, PSS:NICU, Maternal mental health, Breastfeeding

## Abstract

**Background:**

Pump-dependent mothers of very low birth weight (VLBW, < 1500g) infants experience specific challenges achieving sufficient milk supply in the neonatal intensive care unit (NICU) and are therefore less frequently able to achieve (exclusive) breast milk feeding. Stress due to the limitations on participating in the infant’s care may contribute to this problem. Some explorative studies suggest that pressure to provide milk may be an additional stressor in mothers. However, the type of pressure to provide milk perceived by mothers of VLBW infants has rarely been examined.

**Methods:**

A retrospective and anonymous questionnaire was conducted with mothers of VLBW infants aged 6 to 24 months at the time of data collection. Quantitative data and written comments were used to examine the mothers’ perceptions. Descriptive and bivariate tests (Spearman´s rho, Pearson’s chi^2^) were performed to show correlations between pressure to provide breast milk, parental stress (PSS:NICU: role alteration subscale), milk volume, and maternal factors. Pressure to provide milk was measured through two self-developed single items to differentiate between internal and external pressures.

**Results:**

Data of *n* = 533 mothers of VLBW infants was analysed. More than 70% of the mothers agreed that they pressured themselves to provide milk for their infant. In contrast, 34% of the mothers agreed that they felt pressure from outside to provide milk. Higher milk volume 14 days post-partum was significantly correlated with less internal (Spearman´s rho = 0.2017, *p* = 0.000) and less external pressure to provide milk (Spearman´s rho = 0.2991; *p* = 0.000). Higher PSS:NICU parental role alteration scores were significantly correlated with more internal (Spearman´s rho = -0.2865, *p* = 0.000) and more external pressure to provide milk (Spearman´s rho = -0.1478; *p* = 0.002). Milk volume 14 days post-partum and the PSS:NICU were not significantly correlated (Spearman´s rho = -0.0190; *p* = 0.701). Qualitative analyses highlighted these results and enhanced the bidirectional relationships between maternal pressure to provide milk and milk volume.

**Conclusions:**

Especially internal pressure to provide milk is perceived by many mothers, being mutually dependent on milk supply and parental stress. Pressure to provide milk may be an important factor to decrease maternal stress in the NICU and, therefore, lead to more positive pumping and breastfeeding experiences. More research and validated instruments are needed to adequately measure pressure to provide milk with its different psychological, social, and environmental dimensions.

## Background

The first choice of nutrition in preterm infants is mother´s own milk (MOM), followed by donor human milk if MOM is not available [1]. Mothers of very low birth weight (VLBW, < 1500g) infants experience specific challenges when establishing sufficient milk supply in the neonatal intensive care unit (NICU) and are, therefore, less frequently able to achieve (exclusive) feeding with MOM [2, 3]. The infant´s physiological immaturity requires alternative methods of milk expression rather than direct breastfeeding [4]. The impact of preterm birth on secretory activation hinders lactation initiation, which may be further delayed by the mother´s recovery from medical complications during preterm birth and absence of lactation support for mothers, thus contributing to a lower milk supply [1, 5, 6]. Many other factors have also shown to be commonly associated with milk supply and/or (exclusive) breastfeeding among mothers of preterm infants, such as maternal educational level, age, previous children, and experiences with milk expression [7–9].

In addition, mothers of preterm infants are more likely to experience stress, depression, and anxiety than term-mothers, which may be triggered by a traumatic preterm birth, separation from the infant, and limitations in participating in the infant´s care in the NICU [10, 11]. The emergence of stress can be explained as a negative experience arising from distinctions between demands and resources of a person in interaction with his or her environment [12], while parenting stress describes those discrepancies connected to parenthood [13]. Considering stressors due to parenting is particularly relevant, as stress caused by the parental role alteration within the NICU setting, where finding a parent-infant bond through caregiving is hindered, may have a negative impact on maintaining breastfeeding [14, 15]. Moreover, a few explorative studies with term-mothers indicate that pressure to provide milk may be an additional stressor in mothers who want to feed their infants with their own milk [16, 17]. Given the crucial importance of MOM to medical outcomes of preterm infants, the knowledge about this topic could further increase the pressure on mothers to provide milk [18]. Therefore, especially for mothers of preterm infants, the possibility of receiving donor human milk for the infant can reduce negative emotions such as guilt and grief, when they struggle achieving their own milk supply [19]. However, to what extend pump-dependent mothers of VLBW preterm infants perceive pressure to provide milk and how the already named factors are related to it remains rather unclear.

The objective of this study is threefold: First, we aim to measure mothers’ perceptions of pressure to provide milk and illustrate its prevalence among mothers of VLBW infants. Further, factors associated with pressure in providing milk are identified. For this purpose, correlations between the perceived pressure and maternal factors, including educational level, age, previous experience with pumping, milk supply, parental stress, and the availability of donor milk in the hospital are examined. Then, in order to further explore the mother`s perceptions, qualitative data in the type of written comments are assessed.

## Methods

### Study design

Both data were collected through a written, cross-sectional survey. It was part of the Neo-MILK project, which, amongst others, aims to describe the perceptions of preterm mothers regarding lactation and lactation support in the NICU [20]. The study was publicly funded by the Innovation Fund of the Joint Federal Committee (funding code: 01NVF19027) and registered in the German Register of Clinical Trials (ID: DRKS00024799). The study was approved by the ethical committee of the University Hospital Cologne (20–1547).

The retrospective and anonymous survey was conducted in cooperation with four statutory health insurance companies (AOK Rhineland/Hamburg, TK, DAK, Pronova BKK). Data were collected from June to August 2021. The selection criteria were a birth weight less than 1,500 g and an age of 6 to 24 months of the infant at the time of data collection (ICD10 criteria: P07.00, P07.01, P07.02, P07.10, and P07.11.). All mothers who matched these codes and were insured with these companies (1,894) were contacted by their health insurance company and invited to participate in the study. A period of at least six months after birth was chosen for the recruitment of the survey in order to minimize the risk of re-traumatization as studies have shown that post-traumatic stress after preterm birth can last for several months and produce anxiety and depressive symptoms [21, 22].

### Survey instrument and measures

The survey included one subscale of a validated scale and self-developed items based on the current literature.

The perception of pressure to provide milk was divided into two sources of pressure — internal and external pressure — and was measured agreement with two statements. Internal pressure was measured with the following statement: “During the time in the NICU, I pressured myself because I wanted to provide milk for my infant”. External pressure was measured as follows: “During the time in the NICU, I felt pressured from outside to provide milk for my child”. A six-point Likert scale (“totally agree”, “mostly agree”, “rather agree”, “rather disagree”, “mostly disagree”, “totally disagree”) was used for the responses.

Maternal perceptions of stress caused by the limited ability to care for the infant was measured using the parental role alteration subscale of the validated German version of the Parental Stressor Scale: Neonatal Intensive Care Unit (PSS:NICU_German/2scale) [23]. The questionnaire contained only the parental role subscale, as the parental role alteration showed to be the greatest source of stress in parents and the study focuses on the parental caregiving role [24]. Furthermore, since the PSS:NICU_German/2scales score was previously used and reported at the subscale level, we applied it accordingly [25]. In short, it consists of six items, which represent possible stressors and are answered on a five-point Likert scale of “not at all stressful” (1), “a little stressful” (2), “moderately stressful” (3), “very stressful” (4), and “extremely stressful” (5) and the option of “not experienced” (0). The scale can be calculated by two metrics. For the analysis, metric 1 was used, which provides information about the stress occurrence level, meaning stress due to a specific situation. This is calculated by adding all answers on the respective subscale and dividing by the numbers of items. Therefore, higher scores indicate higher levels of stress [23].

For previous experiences with pumping, the options “yes” and “no” were given. To measure maternal milk supply, the milk volume on day 14 post-partum was chosen, as it showed to be a robust indicator for reaching sufficient lactation [3]. Milk volume 14 days post-partum was measured through pre-defined groups of “under 300ml/day”, “301–500ml/day”, “501–700ml/day”, “700–800ml/day”, and “over 800ml/day” (categorial variable). These pre-defined groups were given in order to simplify the mother´s recollection of the milk volume in case she did not document it. The mother’s educational level was measured through pre-defined groups as well, representing the different options for school qualifications in Germany, also including no qualification (categorial variable). Maternal age was openly asked (continuous variable). For the availability of donor milk, the options “Yes”, “No” and “I don´t know” were given. This variable was dichotomised, as it is presumed that if mothers didn´t know about the availability of donor milk, this option was not provided at the hospital.

At the end of the questionnaire, the option was given to enter free texts (“Do you want to tell us more? You can write it here:”). These comments were further analysed, as is described below.

### Data analysis

Quantitative data were analysed using Stata 16. Descriptive data are presented with numbers and percentages in case of categorial variables, whereby continuous variables are presented with means and standard deviations.. To explore and illustrate possible associations with a variety of factors, bivariate tests depending on data distribution (Spearman´s rho, Pearson’s chi^2^/Cramer´s V) between pressure to provide milk, milk volume, previous experience with pumping, parental stress, and demographics were performed on a 95 percent significance level. Therefore, statistical significance of correlations was considered with a p-value equal or less than 5%. Spearman´s-Rho (rs) correlation test was performed in case of ordinal scales and Pearsons-Chi^2^-Test (X^2^)/Cramer´s V in case of binary variables. Correlation coefficients were interpreted as recommended by the literature [26, 27].

Written comments were assessed with an inductive content analysis according to Elo et al. (2008), starting with reading all comments, setting up similar notes for every mentioned dimension, and grouping them into subcategories [28]. Then, subcategories were grouped into two larger categories. This procedure was performed by two persons separately and compared afterwards, creating the results. Quotes from the qualitative data which contained stress or pressure due to providing milk are used to give a wider understanding of the quantitative results.

## Results

In total, 600 of 1,894 mothers participated in the survey, representing a response rate of 31.67%. After data correction of incorrectly contacted mothers, who did not meet the ICD-10 inclusion criteria (*n* = 67) and those who did not initiate lactation (*n* = 15), responses of 518 mothers remained for the following analyses. Characteristics of the participating mothers are presented in Table 1.

**Table 1:** Characteristics of the participating mothers

Characteristics	n (%)
Maternal educational level	
Without a graduation	10 (1,93%)
Lower secondary school	46 (8,88%)
Secondary school	114 (22,01%)
Higher education entrance qualification	124 (23,94%)
University degree	213 (41,12%)
Missing	11 (2,12%)
Previous experience with pumping	
Yes	97 (18,73%)
No	419 (80,89%)
Missing	2 (0,39%)
Milk volume 14 days p.p	
< 300ml/day	196 (37,84%)
301-500ml/day	115 (22,20%)
5001-700ml/day	77 (14,86%)
701-800ml/day	44 (8,49%)
> 801ml/day	68 (13,13%)
Missing	18 (3,37%)
Availability of donor milk at the hospital	
Yes	121 (22,7%)
No	403 (75,61%)
Missing	9 (1,69%)
	(n [mean (min–max)]
Maternal age	487 [34.1 (19–54)]
PSS:NICU (subscale parental role alteration)	427 [3.8 (1–5)]
Cronbach´s alpha	0.8601

### Quantitative results

**Fig. 1:**
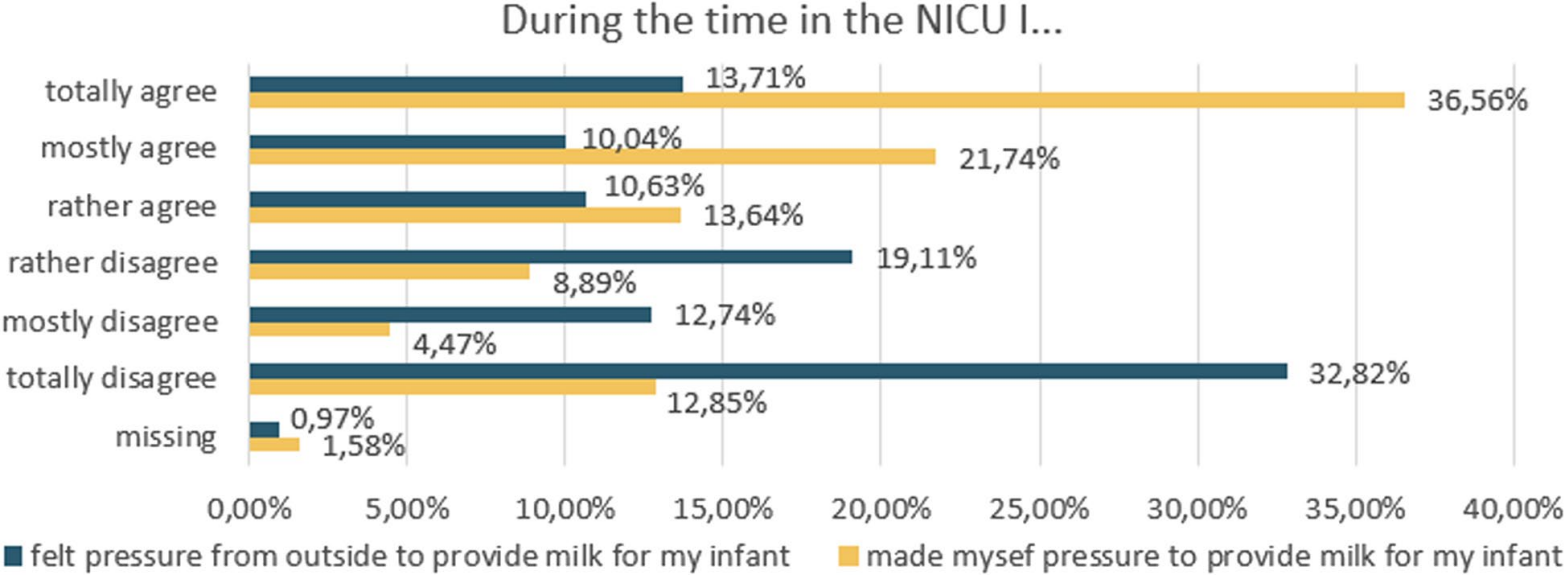
External and internal pressure to provide milk

More than 70% of the mothers totally, mostly, or rather agreed that they pressured themselves to provide milk for their infant (internal pressure). In contrast, 34% of the mothers totally, mostly, or rather agreed that they felt external pressure from outside to provide milk, whereas 33% totally disagreed that they experienced external pressure (Fig. 1).

**Fig. 2:**
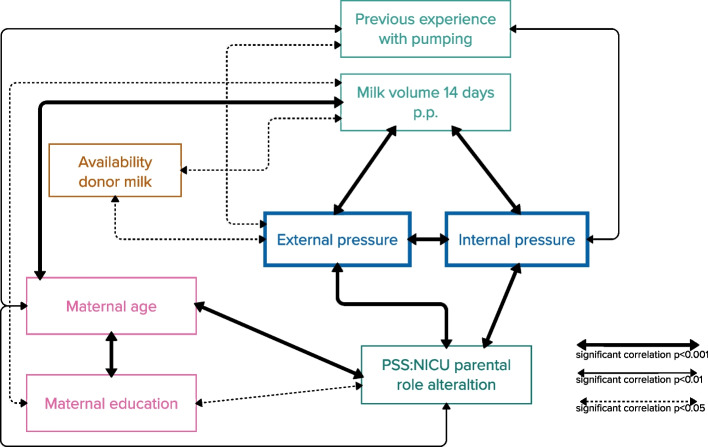
Bivariate correlations

Figure 2 presents the multifaceted correlations between internal and external pressures to provide milk, milk volume 14 days post-partum, maternal demographic factors (education, age), previous pumping experience, and the PSS:NICU parental role alteration subscale. Thicker arrows indicate higher significance, and shorter arrows between the indicators means stronger correlation.

Significant and insignificant correlations between both dimensions of pressure and factors concerning previous experience with pumping, milk volume 14 days post-partum, demographics, and the PSS:NICU subscale parental role alteration can be seen in Table 2. Bold text indicates significance. Internal and external pressure showed a weak to moderate positive correlation (rs = 0.3414; *p* = 0.000). Weak significant, negative correlation appeared between the PSS:NICU and internal pressure to provide milk, where less pressure was correlated with lower PSS:NICU parental subscale scores (rs = -0.2865, *p* = 0.000). Higher milk volume is positively correlated with less internal pressure (rs = 0.2017; *p* = 0.000). Higher levels of parental stress are correlated to higher internal pressure Nevertheless, positive correlations between higher milk volume and less external pressure were stronger (rs = 0.2991; *p* = 0.000) than negative correlations of higher PSS:NICU subscale role alteration scores and higher external pressure (rs = -0.1478; *p* = 0.002).

**Table 2:** Correlations of factors with internal and external pressure to provide milk

Variables	Maternal age	Education	Experience with pumping	Milk volume	PSS:NICU (subscale role alteration)	Availability donor milk
**Internal pressure to provide milk**	rs = 0.0238; *p* = 0.605	rs = 0.005; *p* = 0.904	**X**^**2**^** = 17.398; *p* = 0.004; Cramer ´s V = 0.185**	**rs = 0.2017; *p* = 0.000**	**rs = -0.2865; *p* = 0.000**	X^2^ = 2.111 *p* = 0.834
Maternal age	-	**rs = 0.2363; *p* = 0.000**	**X**^**2**^** = 50.216; *p* = 0.009; K = -0.124**	**rs = -0.1646; *p* = 0.000**	**rs = -0.169; *p* = 0.001**	X^2^ = 40.251 *p* = 0.080
Education	**rs = 0.2363; *p* = 0.000**	-	X^2^ = 0.2023; *p* = 0.975	**rs = 0.0964; *p* = 0.033**	**rs = -0.1085; *p* = 0.027**	X^2^ = 3.971 *p* = 0.410
Experience with pumping	**X**^**2**^** = 50.216; *p* = 0.009; Cramer´s V = 0.322**	X^2^ = 0.2023; *p* = 0.975	-	X^2^ = 7.7170; *p* = 0.103	X^2^ = 32.9691; *p* = 0.082	X^2^ = 0.051 *p* = 0.821
Milk volume 14 days p.p	**rs = -0.1646; *p* = 0.000**	**rs = 0.0964; *p* = 0.033**	X^2^ = 7.717; *p* = 0.103	-	rs = -0.0190; *p* = 0.701	**X**^**2**^** = 10.352 *p* = 0.035; Cramer´s V = 0.145**
PSS:NICU (subscale role alteration)	**Pearson´s Correlation = -0.169; *p* = 0.001**	**rs = -0.1085; *p* = 0.027**	X^2^ = 32.9691; *p* = 0.082	rs = -0.0190; *p* = 0.701	-	X^2^ = 32.671 *p* = 0.087
Availability donor milk	X^2^ = 40.251 *p* = 0.080	X^2^ = 3.971 *p* = 0.410	X^2^ = 0.051 *p* = 0.821	**X**^**2**^** = 10.352 *p* = 0.035; Cramer´s V = 0.145**	X^2^ = 32.671 p = 0.087	-
**External pressure to provide milk**	rs = -0.0134; *p* = 0.769	rs = -0.0434; *p* = 0.332	**X**^**2**^** = 13.047; *p* = 0.023; Cramer´s V = 0.160**	**rs = 0.2991; *p* = 0.000**	**rs = -0.1478, *p* = 0.002**	**X**^**2**^** = 14.410 *p* = 0.013; Cramer´s V = 0.169**

A higher maternal educational level was correlated with lower parental stress (rs = -0.1085; *p* = 0.000). Maternal age and previous experience with pumping were significantly correlated (Cramer´s V = 0.322), but there was no direct significant correlation between maternal age and both dimensions of pressure to provide milk (*p* = 0.605; *p* = 0.769). However, higher maternal age was positively correlated with a higher milk volume 14 days post-partum (rs = 0.1646; *p* = 0.000), although previous experience with pumping was not significantly correlated to milk volume (X^2^ = 7,717; *p* = 0.103). The availability of donor milk was significantly associated with external pressure (X^2^ = 14.410 *p* = 0.013) and milk volume (X^2^ = 10.352 *p* = 0.035), but not with internal pressure.

### Results of qualitative data

In total, comments from 153 mothers were analysed, of which 12 contained information about pressure and/or stress to provide milk. Pressure can be defined as an “excessive or stressful demand” and “often the source of cognitive and affective discomfort” [29] and is, therefore, treated as a term for stress in the analyses. The analysis of the comments resulted in two categories described below.

#### Internal pressure and stress to provide milk

Eight comments concerned pressure and/or stress the mothers placed upon themselves to provide milk. One mother who pressured herself to provide milk also reported high stress levels due to the situation in the NICU, leading to a lower milk supply:

*“"I never felt pressured to breastfeed my child by my surrounding. I put myself under pressure to do so, because it´s the best for my child. [...] My child needed an operation, and out of all the anxiety and fear I had hardly any milk, so I stopped."*

Another mother experienced stress due to a lower milk supply:

*“Pumping was very stressful and a burden for me. The small amount of milk really stressed me.”*

Some mothers explained that they wanted to feed their infant with their milk too much because of the importance and relevance of MOM for the infant:

*"I think, at the end, I put myself under pressure because I wanted it too much."*

*“The importance and impact of mother’s milk became clear to me in the NICU. With this comes responsibility, but also pressure.”*

One mother reported that she did not put herself under pressure, as she already had previous breastfeeding experiences with her first child:

*“I breastfed my first child only three months. Therefore, I didn´t pressure myself with the second child. I was happy about every millilitre, but as it decreased at some point, I accepted it."*

#### External pressure and stress to provide milk

Four comments concerned pressure from outside. The main influences on pressure to provide milk from the environment were the hospital staff and society. Some of these mothers did not want to pump/breastfeed but got pushed by the hospital staff to do so. A few mothers reported psychological issues due to this external pressure:

*“Nurses often put pressure on me that I really needed to breastfeed.”*

*“I was already ‘informed’ (pushed) to breastfeed four days before the caesarean section, daily pushed until two or three days after birth by different persons (physicians, midwives, nurses). [..] The extreme pressure to breastfeed, the anxiety and fear about my children, and my bad psychological condition at the time of admission led to three major breakdowns.”*

*“We received a lot of support with breastfeeding, but I felt under pressure, because I didn´t want to do it. The day I was discharged, I weaned immediately and felt so much better psychologically.”*

Another mother felt pressure to provide MOM by society or the social environment:

*“Meanwhile, it is more of a problem that women who could not breastfeed are treated rather despicably by society. As if they just don't want it enough. There should be more acceptance when women cannot breastfeed for health reasons. I was very bad physically — that's stressful enough — but the pressure from the outside when you don't breastfeed is very stressful.”*

The qualitative data emphasized the interdependency between milk supply, high levels of stress due to the situation in the NICU and internal pressure, which was also reflected the quantitative data. Furthermore, they indicated similar findings on the association between previous experiences with pumping and internal pressure to provide milk. In case of external pressure, the qualitative data expanded the quantitative results by showing the main sources being the NICU staff and the society.

## Discussion

Our data showed that most of the mothers put themselves under pressure to provide milk. In contrast, only one third agreed they were pressured from the outside to provide milk for their infant. This coincides with recent results from Korth et al. (2022), who identified term-mothers themselves as one of the greatest sources of pressure to breastfeed apart from two external sources, which were society and lactation consultants [30]. Our results highlighted the importance of internal pressure to provide milk as a common concern also among mothers of VLBW preterm infants. Nevertheless, external pressure should also be included as an important dimension in the perception of pressure to provide milk.

Internal and external pressures showed to be significantly correlated with each other, which indicated their interrelationship and the importance considering both dimensions of pressure. However, results reveal that some mothers did not experience pressure to provide milk, which has already been observed elsewhere [31]. Mothers with previous pumping experience reported significantly less internal pressure to provide milk for their infant than mothers without pumping experience. The qualitative data highlighted this result, as one mother who reported no feelings of internal pressure related this to her experiences with previous children. In the literature, positive previous experiences with breastfeeding showed to be protective for high breastfeeding self-efficacy and, therefore, higher breastfeeding duration [32]. Huang et. al (2019) elaborated that previous experiences with breastfeeding or milk expression encourage lactation initiation and breastfeeding duration in the actual breastfeeding event [8]. Our results indicated that the positive effect of previous experiences could also be noticeable for a lower internal pressure to provide milk and, therefore, acted like a mediator for milk volume.

Of note, whereas there was a correlation between milk volume and pressure to provide milk, on the one hand, and pressure and the PSS:NICU subscale role alteration, on the other hand, we could not find a significant correlation between milk volume and the PSS:NICU subscale role alteration. In the qualitative data, the stressful situation after preterm birth and low milk volume seemed to be mutually dependent. Stress may negatively affect lactation due to hormonal changes and maternal conditions among preterm mothers [33]. However, there are contrasting findings in the literature, where stress is both associated with lactation and shows no association [34–36]. This may be the case because stress is measured differently in those studies depending on the population of term, late preterm, or low birth weight preterm infants.

The role alteration subscale of the PSS:NICU is primarily intended to depict the situation of the lack of the parental caregiving role [11]. Although the items of the parental role alteration scale include the limited possibility to feed the infant by oneself, this terminology is not further specified. Following the concept of parental stress by Deater-Deckard (1998), the perceived discrepancy between the demand of providing MOM for the infant and the mother´s resources, namely the ability to do so, would depict maternal stress [13]. The only study, to our knowledge, that examined a correlation between stress due to the parental role alteration and milk volume also did not find any significance between those two factors [15]. In our study, the correlation between higher scores on the PSS:NICU subscale role alteration and higher internal and external pressure, and at the same time the correlation between higher internal and external pressure and lower milk volume, indicated that pressure to provide milk could be another important stressor interacting with maternal milk supply. Perhaps that is one reason why hardly any studies have been able to show a correlation between stress within the parental role and milk volume so far, as this factor has not been considered. Indeed, Dowling et. al (2012) and Ikonen et. al (2016) both identified a sufficient milk supply as a frequent concern among milk-expressing mothers of preterm infants [37, 38]. Although there are no studies focusing on pressure to provide milk among preterm mothers, Ayers et al. (2019) identified five categories of stressors in term-mothers, of which one is pressure to breastfeed [17]. Another study with term-mothers elaborated that psychological pressure to breastfeed may have the potential to contribute to postpartum depression, especially in new mothers [16]. Our findings extended these results to suggest that pressure could also be a common concern for preterm mothers, which may be even more triggered by the limited possibilities to care for and feed their infant. In particular, providing milk was described by preterm mothers as equal to “giving life” to their infants and keeping them healthy [39]. Therefore, pressure to provide milk probably should be considered when measuring stress due to the parental role in lactating mothers of preterm infants.

In addition, it should be mentioned that internal pressure was more strongly correlated to scores of the PSS:NICU subscale role alteration than external pressure, whereas external pressure was more strongly correlated to milk volume 14 days post-partum than internal pressure. This emphasises the relevance of including the different dimensions of pressure, as they seem to have various interrelationships with other determinants. However, it must be noted that no directional relationship can be specified for the correlation between internal and external pressure and milk volume or the PSS:NICU subscale role alteration. Moreover, the qualitative data showed that especially internal pressure was indeed triggered by the low milk volume, which underlines their mutual relationship.

Only 23% of the mothers stated that there was a possibility to receive donor milk for the infant. In Germany, human milk banking and usage of donor human milk is not implemented nationwide, for instance due to a lack of consistent legislative frameworks for donor milk [40]. A recent study reported a rather low utilization rate of donor milk with about 35% in their sample of German hospitals [41]. Thus, the knowledge about the possibility and availability of donor milk in general might be low in the German population. In our data, only external pressure was significantly correlated to the availability of donor milk. It might be the case that only mothers, who struggled with achieving milk supply are informed about the alternative of using donor milk and, therefore, felt less pressured from the outside to provide their own milk. Presumably, there might be a more complex association between milk volume, internal and external pressure and the availability of donor milk. However, the data presented here does not allow further conclusions, as the availability of donor milk was not examined in detail. For future research, especially in populations were donor milk is more routinely integrated and more widely known, this might be an important factor to be included.

### Strengths and limitations

The study was strengthened by using quantitative data and qualitative data to more deeply understand the pressure to provide milk among mothers of VLBW preterm infants. In this regard, it must be noted that the qualitative analyses refer to only 12 mentions. Moreover, it can be assumed that the answers are biased, as it is more likely that mothers left comments who had a traumatic or negative experience. Considering that the questionnaire included many topics regarding the time in the NICU, it is indeed surprising that 12 mentions were made regarding stress and pressure. However, a better understanding of this topic may be possible by assessing in-depth interviews with preterm mothers.

Pressure to provide milk was measured through two self-developed single-item measurements, as no validated construct was available at the time of data collection. Even though single items showed to be robust in other studies [42], the qualitative data indicated that there may be more dimensions especially of external pressure to provide milk, which could be evaluated separately in order to define specific sources of external pressure more precisely. Although the analyses showed various correlations between pressure and different variables, there may be many more influencing factors on the pressure to provide milk among mothers of preterm infants, which were not included in this study.

In addition, the retrospective character of the survey may have led to recall bias in the perceptions of mothers. This could have been the case in the retrospective reflection of stress experience during the time in the NICU, as well as in the reproduction of milk volume on day 14 post-partum. Although more than half of the mothers (56%) stated that they had documented their milk volume and did not have to provide information on the basis of memory, the other responses may be biased due to recall. Moreover, it is important to note that the PSS:NICU application was not developed for retrospective surveys due to changes in parental stress experiences over time [23]. Nevertheless, recent research showed robust results with a retrospective use of the PSS:NICU subscale role alteration with a comparable Cronbach´s alpha to our study [25]. Future studies should consider measuring all subscales of the PSS:NICU scale in order to depict more dimensions of stress.

Notwithstanding these limitations, this study provided new insights into the perception of pressure to provide milk as a possible factor being relevant for the pumping and breastfeeding experience, as well as maternal stress levels among mothers of VLBW preterm infants.

## Conclusion

Many mothers of VLBW infants pressured themselves to provide milk, and some feel pressured from outside. Pressure to provide milk may be an important factor to be aware of in order to decrease maternal stress in the NICU. More research and validated instruments are needed to adequately measure pressure to provide milk with its different psychological, social, and environmental dimensions.

## Data Availability

The datasets used and/or analysed during the current study are available from the corresponding author on reasonable request.

## Abbreviations

MOM: Mother´s own milk
NICU: Neonatal Intensive Care Unit
PSSNICU: Parental Stress Scale: Neonatal Intensive Care Unit
VLBW: Very Low Birth Weight
rs: Spearman’s Rho-Test
X^2^: Pearson’s Chi²-Test
