# Correction: Pressure to provide milk among mothers of very low birth weight infants: an explorative study

**DOI:** 10.1186/s12884-024-06374-6

**Published:** 2024-03-04

**Authors:** Isabella Schwab, Till Dresbach, Tim Ohnhauser, Dirk Horenkamp‑Sonntag, Nadine Scholten, Neo-MILK

**Affiliations:** 1 Institute of Medical Sociology, Health Services Research, and Rehabilitation Science, Faculty of Medicine, Health Services Research University of Cologne, University Hospital Cologne, Eupener Strase 129, Cologne, 50933 Germany; 2 Department of Neonatology and Pediatric Intensive Care, Children’s Hospital, University of Bonn, Venusberg‑Campus 1, Bonn, 53127 Germany; 3 Techniker Krankenkasse, Healthcare Management, Hamburg, Germany

**Correction: BMC Pregnancy Childbirth. 2024; 24:134**

10.1186/s12884-024-06315-3.

Following publication of the original article [1], the authors identified errors in the Abstract and the Results section.

The direction of the correlation between milk volume and pressure was incorrectly (reversed) indicated, however the values are correct. The corrections do not change the discussion or conclusion of this publication.

The Abstract and Results section have been replaced with the updated version and changes have been highlighted in **bold text**.

**Abstract**

**Paragraph Results**

In contrast, 34% of the mothers agreed that they felt pressure from outside to provide milk. Higher milk volume 14 days post-partum was significantly correlated with **less** internal (Spearman´s rho = 0.2017, *p* = 0.000) and **less** external pressure to provide milk (Spearman´s rho = 0.2991; *p* = 0.000).

**Results**

**Paragraph 4**

Weak significant, negative correlation appeared between the PSS:NICU and internal pressure to provide milk, where less pressure was correlated with lower PSS:NICU parental subscale scores (rs = -0.2865, *p* = 0.000). Higher milk volume is positively correlated with **less** internal pressure (rs = 0.2017; *p* = 0.000).

The original article has been corrected.
